# Learning dispositions as a lens for teaching socioscientific issues: Attitudes of university biology students towards animal experimentation

**DOI:** 10.1371/journal.pone.0358949

**Published:** 2026-10-16

**Authors:** Jacqueline Dischereit, Susanne Bögeholz

**Affiliations:** Biology Education, Georg-August-Universität Göttingen, Göttingen, Lower Saxony, Germany; National Taiwan Ocean University, TAIWAN

## Abstract

Attitudes towards socioscientific issues (SSIs) represent crucial learning dispositions, as they can influence the students’ willingness to engage with SSIs. Animal experimentation is a particularly contentious SSI, with attitudes ranging from rejection to acceptance. They are influenced by external factors (e.g., research context, test animal) and internal factors (e.g., knowledge, ethical aspects). Understanding these attitudes is essential for fostering reflective engagement with SSIs in higher education. This study (*N* = 503) examines self-reported attitudes of pre-service teacher biology students and non-pre-service teacher biology students towards three contexts: animal experimentation with nonhuman primates, animal experimentation with other test animals, and behavioural research with nonhuman primates. It also explores the internal factors underlying these attitudes. Students’ attitudes differed across contexts: animal experimentation with nonhuman primates was viewed most critically, while behavioural research was perceived most favourably. Pre-service teacher students expressed more ambivalent and negative attitudes than non-pre-service teacher students. Regarding internal factors, a clear explanatory pattern emerged: “pro” attitudes were mainly explained by knowledge, “ambivalent” attitudes by conditions (e.g., the necessity of the research), and “contra” attitudes by ethical aspects. Pre-service teacher students consistently explained their attitudes with more references to knowledge than non-pre-service teacher students. These findings highlight the need for evidence-based integration of learning dispositions into science (teacher) higher education that explicitly addresses the SSI animal experimentation.

## Introduction

Attitudes are central learning dispositions in science education, shaping learners’ motivation and reasoning [1,2]. They are defined as enduring dispositions to evaluate objects, ideas, or situations [1,3]. This evaluative quality distinguishes attitudes from beliefs or opinions [4]. As predictors of intentions and behaviour, they link internal dispositions to decision-making [5]. Attitudes influence how students acquire and apply knowledge and competencies. Thereby, they facilitate or hinder knowledge and competence development [6]. Consequently, research has examined learners’ attitudes towards science and science-related issues [7]. In higher science education, fostering these capacities enables students to critically engage with science-related societal issues. Beyond disciplinary knowledge, students must evaluate evidence, consider ethical implications, and form informed attitudes on controversial socioscientific issues (SSIs). SSIs link science with social and moral dimensions, providing key contexts for developing scientific literacy [8,9], such as socioscientific reasoning and decision-making [10].

In higher education, attitudes and their underlying explanatory patterns, that is, the reasons behind these attitudes, reflect students’ readiness to apply socioscientific reasoning to real-world problems. This represents a key aspect of scientific literacy. Accordingly, higher education must not only convey disciplinary content knowledge but also foster dispositions that enable students to become scientifically literate citizens [11]. The model of educational reconstruction highlights this integration of scientific content and learner dispositions, including attitudes, into university instruction [12,13]. Analysing university students’ attitudes towards SSIs helps inform instructional design and evidence-based, reflective scientific literacy [14].

Animal experimentation exemplifies an SSI at the intersection of scientific progress and animal welfare. It is especially relevant in biology education, where students encounter both scientific methods such as animal experimentation and ethical reflection [15]. Attitudes towards animal experimentation range from rejection to convinced acceptance [15], reflecting its status as a contentious SSI in education and public discourse [16]. The interdisciplinary nature of animal experimentation requires learners to explain their attitude through knowledge as well as ethical and contextual (e.g., societal) considerations [17]. Studies show that animal experimentation is a suitable SSI for educational settings, as learners have some prior knowledge and interest in the topic [16].

A range of external and internal factors has been identified as shaping attitudes towards animal experimentation [17–19]. Among external factors, the research context is often reported as most influential [20]. Support for animal experimentation tends to be higher for less invasive studies and for biomedical research compared with non-medical or non-research use of animals [21]. Environmental or behavioural contexts are often viewed most favourably, yet remain underexplored [19]. Behavioural biology represents a particularly interesting context because it can, but does not necessarily, involve animal experimentation [22], enabling analysis of trade-offs between scientific benefits and animal-welfare concerns.

Another external factor influencing attitudes towards animal experimentation is the type of test animal used [20]. Support declines as the test animals are perceived as more similar to humans or as possessing higher cognitive-emotional capacities [23]. Accordingly, the use of nonhuman primates is associated with most negative attitudes, followed by research using companion animals. Research involving rodents or fish is often viewed as most acceptable [24]. Despite this gradient, explicit comparisons of attitudes towards nonhuman primates and other test animals remain scarce [25,26]. Taken together, research suggests that attitudes towards animal experimentation with nonhuman primates are more negative than towards animal experimentation with other test animals, and that attitudes towards animal experimentation are more negative than towards behavioural research [17,21,23,24].

Beyond external factors, three internal factors are central in shaping attitudes and their explanatory patterns towards animal experimentation. First, knowledge, particularly scientific and procedural understanding, is assumed to support or to explain more differentiated and sometimes more favourable attitudes [21,27]. Some studies indicate a positive association between knowledge about animal experimentation and more positive attitudes [26]. However, this assumption resonates with the ‘deficit model’ [28], which attributes public resistance to controversial scientific topics primarily to a lack of knowledge. This model has been widely criticised, as greater familiarity with an SSI does not necessarily increase acceptance. It may even coincide with lower support when topics such as animal experimentation are perceived as ethically contentious [29].

Second, conditions of animal experimentation, such as the perceived necessity of the research, can explain attitudes [29]. Many people express conditional acceptance, supporting animal experimentation only when animal suffering is minimised or when the purpose is considered justified [30]. This suggests implicit cost-benefit reasoning when forming attitudes towards animal experimentation [31] and indicates that attitudes shift depending on the applied conditions by the person asked.

Third, ethical aspects can explain attitudes towards animal experimentation. Different animal ethics orientations, e.g., animal rights or utilitarianism [32], lead to distinct patterns of acceptance or rejection of animal experimentation [33]. Likewise, views on the moral status or moral treatment of animals shape attitudes [34,35]. For instance, individuals who attribute a lower moral status to animals are generally more accepting of animal experimentation for human benefit [26].

To investigate attitudes towards SSIs, the SEE-SEP model [36] provides a suitable analytical framework. It captures the multidimensionality of SSIs by distinguishing six domains (sociology/culture, economy, environment/ecology, science, ethics/morality, policy) and three aspects of attitude formation (knowledge, value, personal experiences). This model enables the analysis of how students integrate various considerations when explaining their attitudes towards SSIs.

Attitudes towards animal experimentation vary by research context, test animal used, and also by learner group [15,37]. Biology students are especially relevant, as they engage with animal experimentation directly through discussing methods behind scientific advances or animal ethical topics, resulting in some knowledge of the topic [15]. Two learner groups are of particular interest: pre-service teacher (PST) biology students, who will guide pupils’ reasoning on SSIs such as animal experimentation [16], and non-PST biology students, many of whom will conduct or regulate animal experimentation in research and related fields [38]. In many higher education contexts, particularly at the undergraduate level, PST and non-PST students share (biology) courses [39].

PST students are a critical learner group, because their attitudes and explanatory patterns behind attitudes towards SSIs influence willingness to address polarised topics such as animal experimentation and teaching confidence [40]. Research suggests that PSTs reason differently about SSIs than their peers in general biology programs [41]. They express more negative attitudes towards contentious science topics [27]. PSTs are sometimes reluctant to teach SSIs due to limited confidence [9], and negative attitudes have been linked to teaching anxiety in science [42]. Recognising these dispositions and addressing them in higher education can support more confident engagement with SSIs such as animal experimentation in future teaching.

Among non-PST students, attitudes towards SSIs such as animal experimentation influence learning processes and professional decision-making [38]. Since many non-PST biology students will engage directly with animal experimentation, undergraduate education plays an essential role in shaping scientifically and ethically informed perspectives. Educational strategies that foster well-informed and ethically reflective attitudes towards animal experimentation support student engagement and retention [43].

Because PSTs and non-PSTs often learn together but differ in professional orientation, understanding both learner groups’ attitudes is crucial for designing integrative and effective higher education courses [14,44]. Evidence-based insight into attitudinal commonalities and differences can inform curriculum design that prepares students to engage critically with animal experimentation and other SSIs [41].

## Research questions

Previous studies have examined attitudes towards animal experimentation, but systematic comparisons across research contexts, test animals, and different learner groups remain limited [15,37]. Internal factors such as knowledge, conditions, and ethical aspects have rarely been analysed in combination to explain attitudes (explanatory patterns) [18]. Moreover, comparisons of attitudes between PST and non-PST biology students, including the explanations behind them, are scarce. The present study addresses these gaps by analysing self-reported attitudes of PST and non-PST biology students towards three contexts:

- Animal experimentation with nonhuman primates,
- animal experimentation with other test animals, and
- behavioural research with nonhuman primates.

*RQ1a:* What are biology students’ attitudes towards the three contexts?

*RQ1b:* How do attitudes towards the three contexts differ between PST and non-PST biology students?

*RQ2a:* Which internal factors (knowledge, conditions, ethical aspects) underlie biology students’ attitudes towards the three contexts?

*RQ2b:* How do internal factors underlying attitudes differ between PST and non-PST biology students?

## Methods

### Sample

The sample includes 503 undergraduate biology students from five universities across four German federal states. Participants were PST students for secondary schools with biology as a subject, or non-PST biology undergraduates (Table 1). All universities offered comparable teacher education and undergraduate biology programmes, with similar proportions of education-related and subject-specific coursework, ensuring meaningful comparison of PST and non-PST students’ knowledge and attitudes towards animal experimentation.

**Table 1:** Sample composition and characteristics (*N* = 503).

Gender	Percentage	Undergraduate study programme	Percentage
female	63.4%	pre-service teacher biology	42.9%
male	33.6%	non-pre-service-teacher biology	57.1%
diverse	1.4%		
no answer	1.6%		

### Questionnaire

The online survey consisted of three main sections, in addition to socio-demographic questions. Each section addressed participants’ attitudes towards one of the three contexts: animal experimentation with nonhuman primates, animal experimentation with other test animals (than nonhuman primates) and behavioural research with nonhuman primates. After a context description, participants indicated their attitude by choosing one of three attitude categories (“pro”: being in favour, “contra”: being against, or “ambivalent”: having a different attitude). Here, “attitudes” refers to attitudes as self-reported on this closed-ended item with three response options. Then, participants explained reasons for their selected attitude in an open-ended response format. Context descriptions were developed with input from experts in science education, science communication, and animal as well as behavioural research. The three contexts were presented to all participants in the same fixed order, so that the framing of the comparison was held constant across participants. The order was not randomised as the descriptions of contexts were designed to be read in sequence, with animal experimentation with other test animals defined in contrast to the preceding nonhuman primate context.

### Data collection

Data were collected over three consecutive semesters (winter semester 2021/2022 – winter semester 2022/2023) using uniform procedures across all five participating universities. Participants were invited electronically and completed the questionnaire voluntarily. Depending on institutional guidelines, participation was incentivised (€10) or conducted during course time. Prior to data collection, the questionnaire was piloted with *N* = 37 biology students. The piloting focused on the items’ structure (clarity, length) and language (e.g., neutral wording), resulting in minor revisions.

The study was conducted in accordance with relevant local legislation, institutional guidelines, and the guidelines for the social sciences of the Deutsche Forschungsgemeinschaft (DFG, German Research Foundation). Formal approval by the Ethics Commission of the Georg-August-Universität Göttingen was not required for this study. Ethics review for non-medical research in the educational and social sciences is voluntary rather than mandatory at the University of Göttingen. None of the DFG criteria requiring an ethics vote applied because participants were adult university biology students, and the study involved no physical risks, no deception, and no withholding of information. The study complied with the General Data Protection Regulation (EU) 2016/679 and applicable German federal and state data protection law. All participants provided informed consent electronically before participation. They were informed about the study’s purpose, the voluntary nature of participation, their right to withdraw at any time without disadvantage, and their rights under data protection law. Data were stored pseudonymously and were analysed for scientific purposes only.

### Data analysis

We used descriptive statistics to analyse self-reported attitudes towards the three contexts (research questions 1a and 1b). Frequencies and percentages were calculated for each attitude category across the three contexts, as well as separately for PST and non-PST biology students. Inferential analyses were not applied, as the primary objective was to identify overall trends. The closed attitude item identifies which of the three attitudes each participant indicates, not their strength. Differences between animal experimentation contexts and between learner groups are reported as observed distributions within this sample and are not tested for statistical significance. To examine internal factors underlying participants’ attitudes (explanatory patterns behind attitudes; research question 2a and 2b), qualitative analyses were conducted. Rather than measuring these internal factors, we analysed which of them students drew on to explain their attitudes. We followed a coding manual based on the SEE-SEP model [36], which organises reasoning about SSIs into three factors: knowledge, value, and personal experiences. Each of these three factors connects to six categories: sociology/culture, economy, environment/ecology, science, ethics/morality, policy [36]. The model was adapted for this study based on prior applications to animal experimentation (e.g., [16]) and refined in collaboration with a study-accompanying Professional Learning Community, comprising experts in science education, science communication, and animal welfare as well as philosophy [45].

The initial coding manual was developed deductively and included three factors to explain attitudes: knowledge, ethical aspects, and personal experiences. The knowledge factor comprised five categories (sociology/culture, economy, environment/ecology, science, policy), while ethical reasoning was treated as a value-based factor rather than as knowledge of ethics, reflecting the study’s focus on personal ethical aspects. The “personal experiences” factor was restricted to one category, anticipating limited direct experience with animal experimentation among undergraduate students.

No knowledge tests were administered in this study, neither for factual nor for self-rated knowledge. Accordingly, the factor knowledge captures whether students refer to knowledge when they explain their attitudes. It does not refer to how much students know, whether what they refer to is accurate, or how knowledgeable they consider themselves to be. Our results are therefore not informative with respect to the deficit model [28] compared to studies that measured knowledge about animal experimentation directly [21,26]. The results indicate whether students draw on certain knowledge categories (e.g., science, policy) when explaining their self-reported attitude. Our results do not indicate whether knowledge produces a particular attitude.

After the coding manual was piloted with a randomly selected subset of the final dataset (25%, *n* = 126), refinements were made based on emerging response patterns. Theoretical anchor examples were replaced by actual participant responses and inductive (sub-)categories were added. Also, a new factor, conditions, was introduced, as a substantial number of participants explained their attitudes using conditional phrases (e.g., “I am only in favor of animal experimentation if there is no alternative”, ID 392). The final coding manual thus comprised four factors: knowledge, conditions, ethical aspects, and personal experiences. The “personal experiences” factor was excluded from further analysis due to its low response frequency.

All 503 responses were coded by the first author using the final manual (S1 Appendix). A second rater, trained on the final manual but not involved in its development, independently coded a separate, randomly selected 25% subset of the data (*n* = 126 responses). Interrater reliability was quantified using Cohen’s *κ* and was good (*κ* = .84). All discrepancies were resolved through discussion until consensus was reached. Exemplary statements and corresponding codes are presented in Table 2.

**Table 2:** Excerpt from coding manual with factors, two exemplary categories per factor, coding rules and corresponding anchor examples for analysing reasons underlying attitudes towards animal experimentation.

Factors	Categories (Subcategories)	Coding rules of categories: Statements are coded…	Anchor examples
Knowledge	Science knowledge (Scientific findings)	... that refer to knowledge or evidence (from the fields of STEM or medicine).	“Animal experimentation is nec[es]sary in order to do research on organ systems and physical reactions to possible medication/vaccines.” (ID 57)
	Policy knowledge (3R principle)	... that refer to political or legal guidelines, regulations, and laws.	“Animal experimentation should always follow the 3R rule (Reduce, Refine, Replace).” (ID 28)
Conditions	Necessity of research	... that address attitudes depending on the perceived necessity of the research.	“[...] As long as experiments are necessary, they should be allowed.” (ID 219)
	Effects of research on animals	... where the attitude depends on the effect on the test animals (pain, suffering, harm) because of their use in the research.	“If no animal had to suffer or gets hurt, animal experimentation would be morally acceptable.” (ID 146)
Ethical aspects	Moral status of animals (Equal moral status humans-animals)	... that ascribe the same moral status to humans and animals.	“Humans are not above animal[s] in any way, shape or form.” (ID 25)
	Moral treatment of animals (Not harming animals)	... that generally reject or prohibit the infliction of pain, suffering, harm on animals.	“No animal should be harmed.” (ID 416)

## Results

### Biology students’ attitudes towards animal experimentation

Biology students’ attitudes towards animal experimentation vary by the external factors research context and test animal used (Fig 1). When animal experimentation involves nonhuman primates, negative attitudes are most frequent (37.4%), followed by positive (32%) and ambivalent attitudes (30.6%). In contrast, attitudes towards animal experimentation with other test animals are more favourable: 37.8% of students express positive views, 32.8% are ambivalent, and 29.4% oppose such research. These distributions indicate that attitudes towards animal experimentation differ by the test animal used, with nonhuman primates showing greater opposition than other test animals.

**Fig 1:**
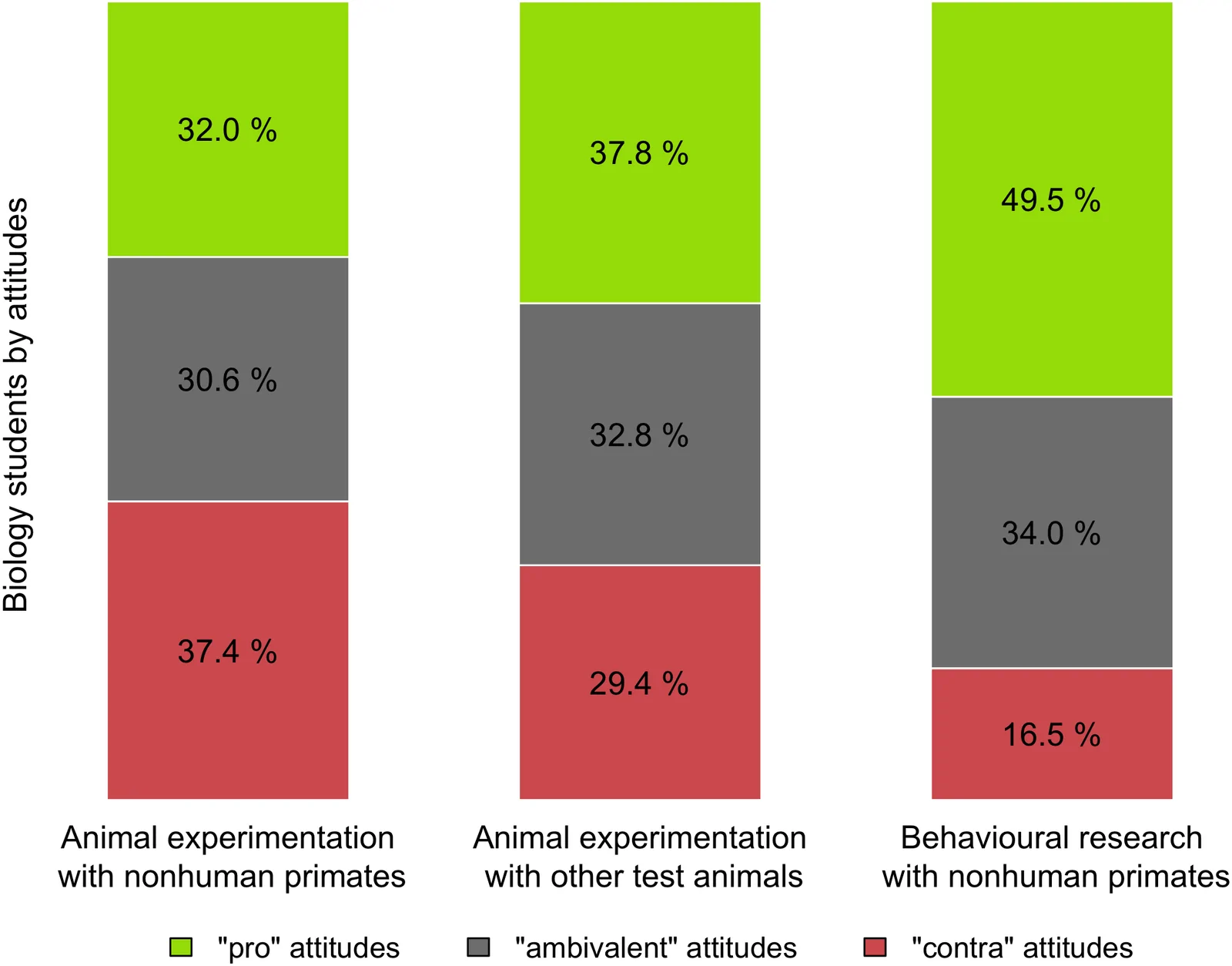
Biology students’ attitudes towards the three contexts (*N* = 503). Bars show proportions of students expressing “pro”, “ambivalent”, and “contra” attitudes towards each context. Note. Percentages refer to all 503 students and sum to 100% within each context.

Across contexts, biology students expressed more negative attitudes towards contexts explicitly involving animal experimentation compared to behavioural research with nonhuman primates. Behavioural research can but does not necessarily involve animal experimentation. Nearly half of all students (49.5%) express positive attitudes towards behavioural research with nonhuman primates, while only 16.5% express negative attitudes. In this sample, biology students were thus more critical of animal experimentation than of behavioural research.

### Differences in attitudes of pre-service teacher and non-pre-service teacher biology students towards animal experimentation

The overall pattern of attitudes across the three contexts (Fig 1) is mirrored when considering PST and non-PST biology students separately (Fig 2). In both groups, animal experimentation with nonhuman primates elicits most negative attitudes, whereas behavioural research with nonhuman primates evokes most positive attitudes.

**Fig 2:**
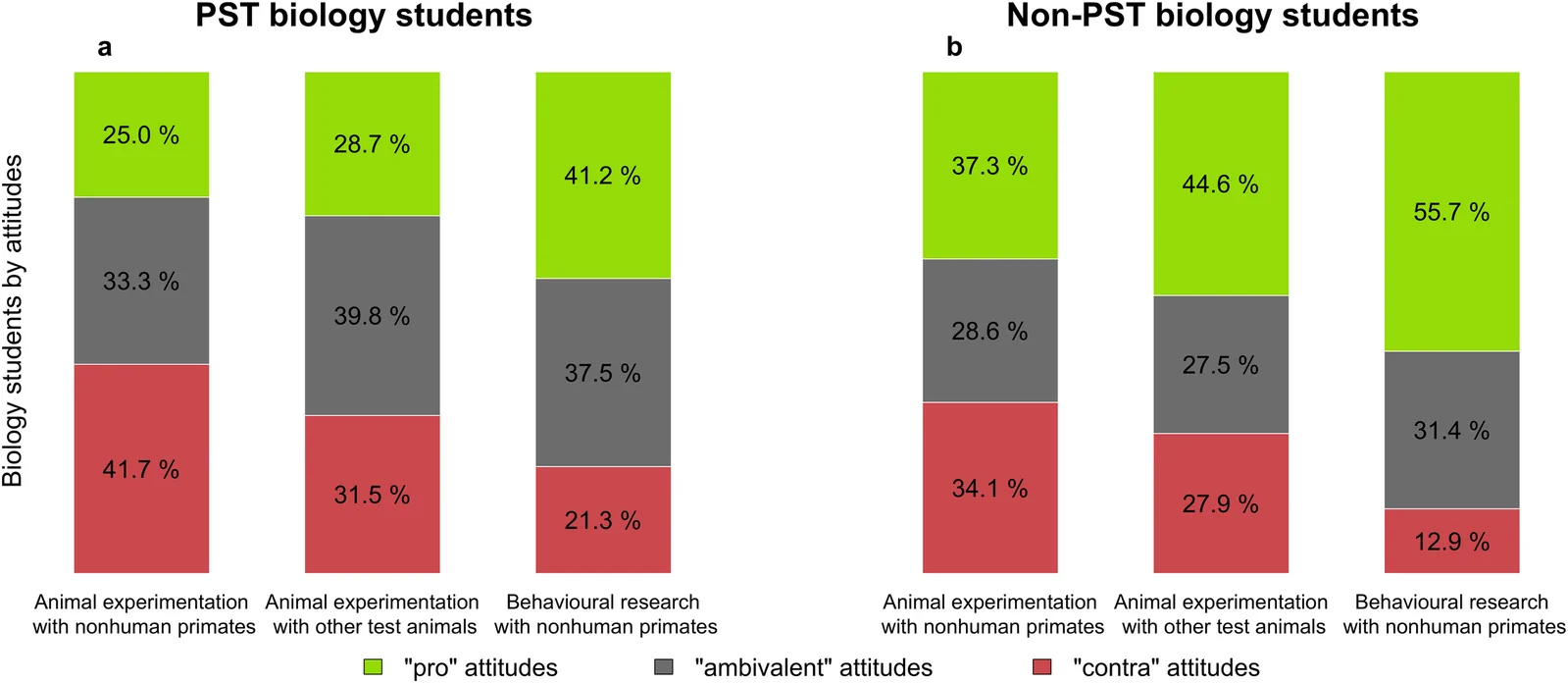
PST biology students’ (2.a, *n* = 216) and non-PST biology students’ (2.b, *n* = 287) attitudes towards the three contexts. Bars show proportions of students expressing “pro”, “ambivalent”, and “contra” attitudes towards each context. Note. Percentages refer to 216 PST (2.a) and 287 non-PST (2.b) students and sum up to 100% within each context.

However, differences emerge in the prevalence of “ambivalent” attitudes. PST students exhibit higher levels of ambivalence across contexts than non-PST students. Among PSTs, ambivalence is most pronounced for animal experimentation with other test animals (39.8%, Fig 2a), while non-PSTs show the lowest level of ambivalence towards this context (27.5%, Fig 2b) but the highest towards behavioural research (31.4%, Fig 2b).

Overall, negative attitudes were descriptively more frequent among PST students than among their non-PST peers, who predominantly held more positive attitudes across contexts. Only for behavioural research does a relative majority of PST students express support (41.2%, Fig 2a). Yet, even in this context, their attitudes remain less positive than the attitudes of non-PST students, who show 12% to 16% more “pro” attitudes towards the three contexts. As set out in the Methods, these differences are reported as observed distributions within this sample.

### Factors underlying biology students’ attitudes towards animal experimentation

Biology students’ “pro”, “ambivalent”, and “contra” attitudes towards the three research contexts are explained by reference to three internal factors: knowledge, conditions of animal experimentation, and ethical aspects. Although their relative prevalence varies across contexts and attitude categories (“pro”, “ambivalent”, “contra”), clear explanatory patterns emerge when analysing which internal factors dominate each attitude category (Fig 3).

**Fig 3:**
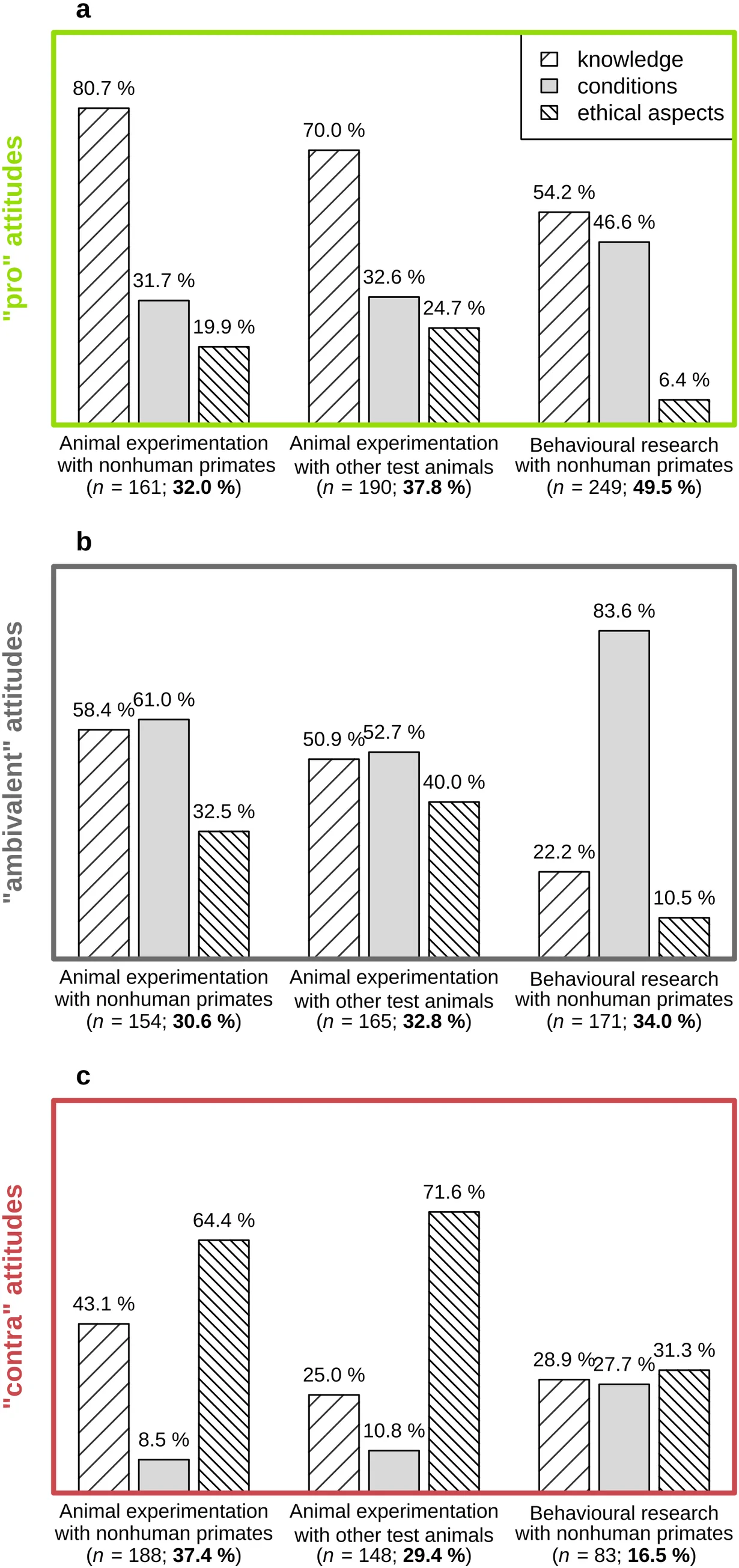
Biology students’ use of internal factors (knowledge, conditions, ethical aspects) explaining “pro” (3.a), “ambivalent” (3.b) and “contra” attitudes (3.c) towards the three contexts (%). Bold percentages vertically add up to 100% of participants per context (cf. Fig 1, *N* = 503). Percentages above bars relate to the number of students per attitude category (“pro”, “ambivalent”, “contra”) and context. Participants who explained their attitudes with more than one internal factor are scored for each factor within one context per attitude category.

Across contexts, biology students expressing “pro” attitudes primarily refer to knowledge to explain their attitudes, followed by conditions and, less frequently, ethical aspects (Fig 3a). For instance, in animal experimentation with nonhuman primates, 80.7% of biology students explain their “pro” attitudes through references to knowledge (e.g., “it might be one of the only effective ways to gather proper data in certain fields, which can then be used to design new methods, to treat diseases or even prevent them”, ID 7). Only 19.9% explain their “pro” attitude with references to ethical aspects. Interestingly, across all three attitude categories, animal experimentation with nonhuman primates is the context most strongly connected to knowledge.

“Ambivalent” attitudes are most frequently explained by conditions, with knowledge categories playing a secondary role (Fig 3b). Similar to “pro” attitudes, ethical aspects are least often connected to ambivalence. For example, among students reporting “ambivalent” attitudes towards behavioural research with nonhuman primates, 83.6% reference conditions in the explanations for their attitudes (e.g., “it is totally fine if it’s about observing animals in their natural habitat […] but I do not think it is okay to put an animal under profuse stress or harm for research purposes”, ID 338). Only 10.5% refer to ethical aspects when explaining their “ambivalent” attitudes. Behavioural research with nonhuman primates shows the strongest association with conditions and the weakest with ethical aspects across all three attitude categories.

In contrast, “contra” attitudes are primarily explained through ethical aspects across contexts, followed by references to knowledge, with conditions playing the least influential role (Fig 3c). For instance, 71.6% of students attribute their “contra” attitude towards animal experimentation with other test animals to ethical aspects (e.g., by ascribing equal moral status to all animals: “just like nonhuman primates, every other animal is a sentient being too and doesn’t deserve to suffer”, ID 468), while only 10.8% reference conditions. Animal experimentation with other test animals is most connected to ethical aspects across all three attitude categories in our study.

Across contexts, 62 out of 503 students (12.3%) referred to knowledge claims that conflict with scientific or legal frameworks, coded as “alternative knowledge conceptions”. These were least common among students reporting “contra” attitudes in each context (0.4%–1.4% of all students) and most common among students reporting “ambivalent” attitudes across contexts (0.5%–3.6%). Most of the 62 students assumed that animal experimentation still serves cosmetics testing (8.9%, e.g., “I am against animal experimentation as they always only use it to test cosmetics”, ID 58). Such statements contradict EU laws banning cosmetic testing (e.g., Regulation (EC) No 1223/2009). The remaining students (3.4%) mostly assumed that the research pursues purposeless ends (e.g., “experiments on nonhuman primates are conducted solely out of a ‘thirst for knowledge’, without any specific purpose or scientific goal”, ID 474), although animal experimentation in the EU requires an approved scientific purpose and a harm-benefit assessment (Directive 2010/63/EU).

Generally, the internal factors behind biology students’ attitudes follow consistent explanatory patterns: reported “pro” attitudes are primarily explained by knowledge, “ambivalent” attitudes by conditions, and “contra” attitudes by ethical aspects.

### Differences in internal factors underlying pre-service teacher and non-pre-service teacher biology students’ attitudes

The overall explanatory patterns for “pro”, “ambivalent”, and “contra” attitudes (Fig 3) generally apply to non-PST students across contexts (Fig 4d–4f). PST students, however, show partly different patterns (Fig 4a–4c).

**Fig 4:**
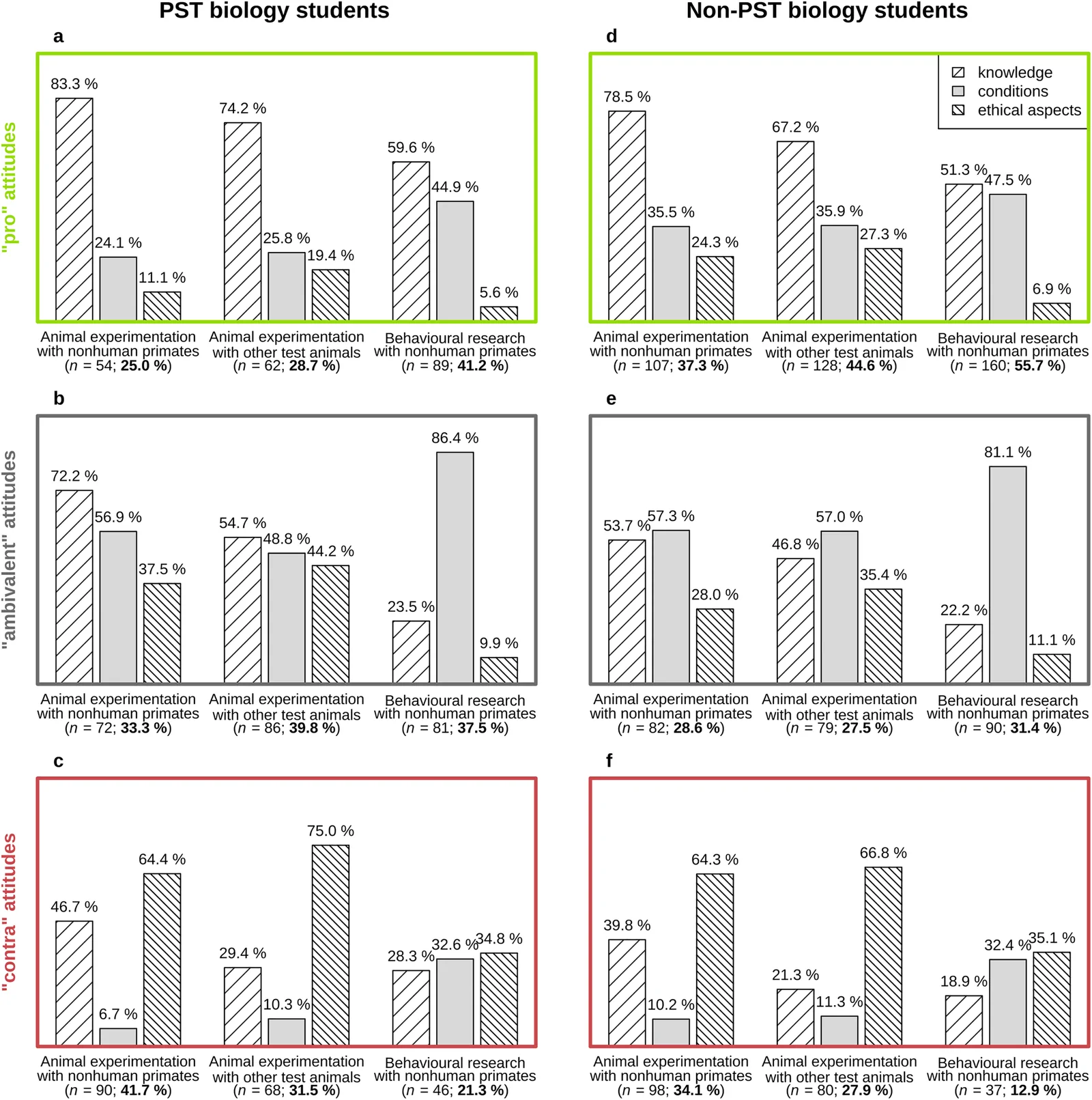
Pre-service teacher (PST) biology students’ (*n* = 216) and non-PST biology students’ (*n* = 287) use of internal factors (knowledge, conditions, ethical aspects) explaining “pro” (PST students 4.a; non-PST students 4.d), “ambivalent” (PST students 4.b; non-PST students 4.e) and “contra” attitudes (PST students 4.c; non-PST students 4.f) towards the three contexts (%). Bold percentages vertically add up to 100% of participants per context (cf. Fig 2). Percentages above bars relate to the number of students per attitude category (“pro”, “ambivalent”, “contra”) and context. Participants who explained their attitudes with more than one internal factor are scored for each factor within one context per attitude category.

For both, PST and non-PST students, “pro” attitudes are mainly explained by knowledge, followed by conditions and ethical aspects (Fig 4a, 4d). Yet, PST students rely more on knowledge-related explanations and less on conditions and ethical aspects when explaining their attitudes.

A key difference between PST and non-PST students emerges in their explanation of “ambivalent” attitudes. Non-PST students mainly attribute their ambivalence to conditions across contexts (Fig 4e), whereas PST students draw more on knowledge – at least for contexts explicitly involving animal experimentation. Despite these differences compared with non-PST students, ethical aspects remain the internal factor least frequently referenced by both groups when explaining “pro” and “ambivalent” attitudes.

For “contra” attitudes in contexts explicitly involving animal experimentation, both learner groups refer predominantly to ethical aspects across contexts, followed by knowledge, with conditions playing the smallest role (Fig 4c, 4f). In behavioural research, both groups reference conditions more frequently than knowledge. Still, PST students refer to knowledge more often than non-PST students when explaining their “contra” attitudes.

In summary, the overall explanatory patterns identified for attitudes apply to non-PST students, while PST biology students display slight deviations. Above all, PST students rely more strongly on knowledge across contexts and attitude categories (“pro”, “ambivalent”, “contra”) than non-PST students when explaining their attitudes.

## Discussion

Understanding learning dispositions of university students is essential for fostering scientific literacy, as they influence how learners evaluate SSIs. Attitudes towards the SSI of animal experimentation provide insights into such dispositions, as they integrate external and internal factors for attitudes (e.g., knowledge, conditions, ethical aspects).

### Attitude patterns concerning animal experimentation

Biology students’ attitudes varied with research context and test animal, indicating that attitudes differed by both external factors (Fig 1). The contrast between the two nonhuman-primate contexts is informative, as they involve the same test animals but differ in research context. This difference in students’ responses reflects that animal experimentation is perceived as more invasive and harmful to the animal than behavioural research: behavioural research is often viewed as less intrusive and therefore more acceptable [17,30]. In line with harm-benefit reasoning models, support for animal experimentation decreases as perceived harm increases [19,46].

Interestingly, behavioural research with nonhuman primates elicited 17.5% more positive attitudes than animal experimentation with nonhuman primates (Fig 1), despite being defined as a context that can, but does not necessarily, involve animal experimentation [22]. This suggests that students associate behavioural research with less invasive methods and lower ethical concern [19]. Thus, framing and perceived purpose of research can influence attitudes, sometimes more than the factual inclusion of animal experimentation.

Compared to animal experimentation with nonhuman primates, experimentation with other test animals was viewed as less critical, consistent with findings that acceptance decreases with increasing perceived sentience or human-likeness of the species involved [15]. In line with Batt [23], this suggests that moral concern is shaped by perceived bio-behavioural similarity to humans, explaining why participants describe primate research as “most difficult to justify” (ID 86). At the same time, studies reporting stronger opposition to experiments involving companion animals indicate that moral concern is also influenced by species familiarity [47]. In the present study, these animals were intentionally grouped (“other test animals” comprised the five most common test animals of 2020: mice, rats, fish, rabbits, birds). This enables a focused comparison with nonhuman primates, but may have obscured distinctions among individual species [17]. Yet, animal experimentation with nonhuman primates elicited most negative attitudes, underscoring their unique ethical status [25].

Notably, animal experimentation with other test animals descriptively elicited more “contra” attitudes than behavioural research with nonhuman primates. This could indicate that the research context exerts a stronger influence on attitudes than the test animal used. This aligns with prior findings suggesting that perceived purpose and invasiveness of research can outweigh species differences in attitude formation [20,26]. The interaction of these external factors highlights the importance of providing students with context-specific opportunities to form attitudes on animal experimentation [17].

Overall, students seem to engage in an implicit cost-benefit calculus, weighing perceived research purpose against potential harm to test animals. Compared with international datasets (e.g., [24]), attitudes in this sample were somewhat more favourable towards animal experimentation, a pattern also found in recent German studies on attitudes and animal ethics orientations regarding animal experimentation [48]. Our results possibly reflect the strong emphasis on methodological rigour and ethical regulations of animal experimentation in German higher education [49].

### Ambivalence as integrative reasoning

A relatively large proportion of participants in this study (31–34% across contexts, see Fig 1) reported “ambivalent” attitudes, exceeding proportions documented in previous research where neutral attitude options were available (21.7% [15]; 23% [24]). Together with findings of even higher proportions of “reserved” respondents (50% [31]), this may point to increasing ambivalence in attitudes towards animal experimentation. This may reflect growing recognition of animal sentience and, within socioscientific reasoning frameworks, signal learners’ efforts to reconcile competing epistemic and moral considerations [50].

From the perspective of the model of educational reconstruction [12], these “ambivalent” attitudes represent productive learning dispositions, indicating, for instance, students’ ability to consider multiple perspectives. The higher prevalence of ambivalence among PST compared to non-PST students is consistent with this interpretation, as socioscientific reasoning is explicitly fostered in German teacher education. This interpretation rests on the students’ explanations rather than on the attitude category alone: across the three contexts, students with “ambivalent” attitudes explained them predominantly by reference to conditions (52.7–83.6%, Fig 3b). More precisely, they specified the circumstances under which their evaluation of animal experimentation would change (e.g., “[...] As long as experiments are necessary, they should be allowed.”, ID 219). Naming such conditions indicates a weighed judgement, though not the resolution of the factual questions the conditions turn on. We cannot exclude that some ambivalence reflects insufficient information. This unresolved remainder is, however, characteristic of SSIs rather than a deficiency in the reasoning. Based on our results, ambivalence is therefore better viewed not as indecision but as evidence of integrative reasoning.

### Patterns in internal explanatory factors

The three explanatory patterns for “pro”, “ambivalent” and “contra” attitudes summarised in Fig 3 were consistent across contexts. This mirrors findings by Lund et al. [31], who reported that “approvers” (comparable to “pro” attitudes) emphasised human benefit. This maps onto our knowledge category, where students explained “pro” attitudes with scientific and medical benefits of animal experimentation. In the same study, “disapprovers” (“contra”) focused on animal cost, which corresponds to the ethical aspects that dominated explanations of “contra” attitudes here, while “reserved” participants (“ambivalent”) alternated between both benefit and cost depending on context.

The association between self-reported “pro” attitudes and knowledge-based explanations aligns with recent findings [21,27]. Rather than supporting a deficit-model interpretation, this suggests that students with positive attitudes draw on scientific knowledge to justify their stance, reflecting trust in scientific expertise and the perceived benefit of animal experimentation, particularly in biomedical primate research [18,25].

Self-reported “ambivalent” attitudes centre on conditions, reflecting students’ attempts to balance ethical and scientific perspectives. This pattern reflects context-dependent attitude formation and aligns with previous findings, where support for animal experimentation depends on minimised animal suffering or clearly justified research purposes, such as treating serious diseases [30]. Such reasoning suggests an implicit cost-benefit evaluation, complicated by ambiguities over what constitutes “justified” research [31]. In our study, behavioural research with nonhuman primates is therefore most closely linked to conditions, likely due to its perceived lower invasiveness and definitional ambiguity regarding whether it includes animal experimentation.

Self-reported “contra” attitudes towards animal experimentation are primarily explained by ethical considerations, with students prioritising animal welfare over potential scientific benefits. This aligns with research showing that differing animal ethics orientations influence how people evaluate the moral status of animals and the legitimacy of human use of animals [32,33]. How strongly students draw on ethical consideration may itself reflect differences in the perceived moral status of animals and human-animal similarity. As smaller or less cognitively complex species (e.g., rodents) are commonly viewed as ‘standard’ laboratory animals [24], moral reflection focused on animal welfare rather than scientific benefit.

Notably, only a small share of explanations of “contra” attitudes rested on premises conflicting with current law or scientific practice, which concerned cosmetics testing almost exclusively. Rejection of animal experimentation in this sample was therefore grounded far more in ethical considerations than in factual misinformation. This aligns with findings that animal ethics orientations, rather than knowledge deficits, shape how people evaluate the moral legitimacy of animal use [32,33], and with reviews describing public opposition as principled rather than uninformed [17]. This underlines that, where an attitude rests on an incorrect premise, that premise is a legitimate starting point for science teaching or science communication. However, it cannot be assumed that correcting such a premise changes the attitude. Informal reasoning on SSIs is not determined by content knowledge alone [50] and deficit-model assumptions about the knowledge-attitude link have been widely criticised [28].

The emergence of “conditions” as one of three explanatory factors behind attitudes extends the SEE-SEP framework [36] by capturing the situational contingencies characteristic of SSIs such as animal experimentation [29]. Students’ explicit reference to such conditions indicates reflective engagement and supports claims that attitudes towards animal experimentation are “nuanced, with evidence of conditional acceptance” [17, p. 166]. Together, these findings point to different reasoning profiles when explaining “pro”, “ambivalent”, and “contra” attitudes: rational-instrumental, contextual-conditional, and moral-evaluative reasoning. This triad merits further investigation in biology education research.

### Differences between pre-service teacher and non-pre-service teacher biology students

Comparing PST and non-PST biology students reveals how disciplinary education shapes attitudes towards animal experimentation (Fig 4). Consistent with Brown et al. [27], the attitudinal differences between the two groups may relate to the limited role of animal experimentation in teacher education. This results in fewer points of contact and less perceived relevance. Previous studies indicate that greater familiarity with or involvement in animal research is associated with more accepting views (e.g., [15]). Thus, limited exposure among PSTs may contribute to their more critical or uncertain stance. Additionally, teacher education often foregrounds ethical reflection and animal welfare, which may further reinforce potential scepticism towards animal experimentation.

PST students’ reported greater ambivalence also reflects curricular emphasis. PSTs are explicitly trained to consider multiple perspectives and to model reflective judgment for their future pupils [10]. In contrast, non-PST students’ training is more closely linked to scientific practice, where animal experimentation may be framed as methodologically necessary and therefore more justified. This aligns with findings that science majors’ learning environments foster trust in scientific rationality [44], while offering fewer opportunities to discuss SSIs [11].

In line with Sorensen et al. [41], PST students explain their attitudes differently than non-PST students. Interestingly, PST students’ more frequent knowledge-based explanations coincide with less positive attitudes (Fig 4). Although studies argue that prior knowledge predicts the types of arguments constructed regarding SSIs [51], this does not indicate greater factual knowledge among PST students, which was not measured here. Instead, our results may reflect pedagogical training that emphasises scientific knowledge as an epistemic resource for balanced moral reasoning [52]. Non-PST students, by contrast, emphasised conditions such as research necessity, reflecting more research-oriented explanations for their attitudes.

Contrasting explanatory patterns of attitudes towards animal experimentation between PST and non-PST students point to distinct but potentially complementary professional orientations in how each group reasons about animal experimentation. Recognising these differences is essential for higher education. Integrated courses of PST and non-PST biology students could foster productive “epistemic exchanges” [53], encouraging dialogue between ethical and scientific reasoning in addressing complex SSIs such as animal experimentation.

## Limitations and future research

The study utilised a sample from five German universities, which may constrain generalisability due to specific cultural and curricular characteristics. Nonetheless, prior research indicates that core animal ethics orientations are conceptually similar across European contexts [54]. The descriptive, cross-sectional design of the present study enabled initial exploration of self-reported attitudes and explanatory patterns but precludes causal inference.

The present study also precludes interpretable inferential comparison between contexts or learner groups, because the fixed presentation order confounds context with presentation position. The three animal experimentation contexts were presented in a fixed order, so carry-over effects cannot be excluded. Therefore, future research should randomise the order of contexts. Moreover, attitudes were captured only with a single forced-choice item for the self-reported “pro”, “contra” and “ambivalent” attitudes, rather than with an established, validated attitude scale. These limitations, the fixed-order presentation and the single-item measurement of attitudes, need to be addressed in a design intended for inferential comparison.

When interpreting the results of the present study, the ambiguous definition of behavioural research (which can but does not necessarily involve animal experimentation) [22] has to be taken into account. It may have introduced interpretive variation among the students, so the corresponding responses should be interpreted cautiously. Our qualitative analyses, however, suggest this had little impact on the results.

While both closed items (for self-reported “pro”, “ambivalent”, and “contra” attitudes) and open-ended items (explanations for self-reported attitudes) were employed, the data primarily capture explicit reasoning; emotional and tacit aspects will be analysed in future work. The adapted SEE-SEP framework, modified by adding “conditions” and omitting “personal experiences”, limits comparability with previous applications (e.g., [16]). However, adding conditions enhanced contextual fit and shed light on the educationally important category of “ambivalent” attitudes towards animal experimentation. Such “ambivalent” attitudes can include informed, ethically reflective, and differentiated stances that weigh conditions, such as specific benefits of animal experimentation (e.g., “I am in favour of animal experimentation as long as it benefits humanity in a meaningful sense, ...”, ID 134). Thus, learning dispositions such as “ambivalent” attitudes towards animal experimentation can, instead of documenting indecisiveness, express a high degree of socioscientific reasoning.

Also, longitudinal and qualitative approaches would allow investigation of implicit and emotional dimensions of attitudes and their development over the course of higher education. Given ongoing scientific findings, e.g., on animal cognition, or shifting social debates [17], longitudinal and further cross-cultural studies would be valuable for examining how emerging knowledge and societal discourse (re-)shape student attitudes towards animal experimentation.

## Implications for research and practice in science teacher and science education

This study advances both the theoretical understanding of self-reported attitudes towards SSIs in higher education and their practical implications for science (teacher) education. Results demonstrate that attitudes are explained by external and internal factors, and that academic pathways influence how students explain their attitudes towards the SSI animal experimentation. PST and non-PST biology students represent distinct learner groups, suggesting that shared learning environments should be designed to promote epistemic exchange and mutual understanding [53]. Consistent with the model of educational reconstruction, attitudes function as learning dispositions that facilitate or hinder knowledge and competence development [6,13]. Understanding students’ attitudes should thus be a default position when teaching SSIs such as animal experimentation [15].

A promising pedagogical framework to bridge various attitudes of different learner groups is the “Two-Eyed Seeing” approach [55]. It encourages learners to view SSIs through scientific, ethical, and holistic lenses, recognising the value of each. The “Two-Eyed Seeing” approach is particularly suited to interdisciplinary higher education contexts. Further, it can inform education by providing future teachers with strategies to guide SSI discussions in schools. For example, students may be asked to articulate their attitudes using scientific evidence, ethical aspects, as well as cultural considerations and to reflect on how these perspectives interact (e.g., through guided discussions or reflective writing tasks).

By extending the SEE-SEP framework with the factor “conditions” for explaining attitudes towards animal experimentation, this study captures contingencies of socioscientific reasoning and identifies “ambivalent” attitudes as productive learning dispositions [31]. These analytical refinements advance a multidimensional study of attitudes and offer a transferable approach for other SSIs such as gene editing or issues in the context of land-use change.

Ultimately, these findings highlight the need to embed evidence-based knowledge of learning dispositions into the design, conduct, and reflective practice of biology education. In particular, greater attention to learners’ attitudes and the explanatory patterns behind them is required, as these shape how students engage with SSIs. Learners’ attitudes and their explanatory patterns can be addressed through targeted educational approaches, especially when teaching learner groups from diverse disciplinary backgrounds.

## Conclusions

Biology students’ self-reported attitudes towards animal experimentation varied with the research context and test animal, while their explanations differed by attitude. “Pro” attitudes were mainly explained by knowledge, “ambivalent” attitudes by conditions, and “contra” attitudes by ethical aspects. Descriptively, PST students more often expressed “ambivalent” or “contra” attitudes and referred more frequently to knowledge when explaining their attitudes than non-PST students.

These findings show that attitudes towards animal experimentation are not uniform, even among students who share the same disciplinary setting. The findings also support treating ambivalence as a distinct position shaped by the conditions under which animal experimentation is considered acceptable. For higher biology education, this suggests that teaching should make space for knowledge-based, condition-based, and ethical reasoning.

The present study provides a first, exploratory account of biology students’ attitudes as learning dispositions towards animal experimentation in higher education, together with the explanatory patterns behind them. Future research could examine (i) these attitudes in more detail, for instance through case-based approaches, and (ii) how they develop over time.

## Supporting information

S1 Appendix Coding manual for internal factors of attitudes towards animal experimentation with nonhuman primates, animal experimentation with other test animals, and behavioural research with nonhuman primates. (PDF)
