# Supplementary material for: Learning dispositions as a lens for teaching socioscientific issues: Attitudes of university biology students towards animal experimentation
Source: PLoS One. 2026 Oct 16;21(10):e0358949. doi: 10.1371/journal.pone.0358949 (PMC13674764; doi:10.1371/journal.pone.0358949)
Supplement: Supplementary file 1 [file pone.0358949.s001.pdf]

Supporting information

S1 Appendix. Coding manual for internal factors of attitudes towards animal experimentation with nonhuman primates, animal experimentation with other test animals, and behavioural research with nonhuman primates

Note: In the following, we abbreviate the terms animal experimentation (AE) and nonhuman primates (NHPs).

<u>Coding rules:</u>

- Coding units are at least complete sentences. If coherent information is described in more than one sentence, the coding unit is formed according to content sections.
- Multiple coding within a sentence is possible.
    - Example: *"Because nonhuman primates are so closely related to humans, the data obtained in animal studies with them [NHPs] are much more transferable to humans."* (ID 9) [CODE 1: similarity between humans & NHPs]; [CODE 2: higher transferability for AE with NHPs]
- The same code can be assigned more than once within one answer. This applies only if the statements can be assigned to the same code but differ in their content-related focus.
    - Example: "*Primates are very similar to humans, and highly intelligent beings.* [CODE 1: similarity humans & NHPs; content-related focus: *highly intelligent beings*] *Their lifespan is much longer than other animals used in animal experimentation. [...] However, since they [NHPs] are so closely related to humans, research on them is often times directly transferable to humans*." (ID 6) [CODE 2: similarity humans & NHPs; content-related focus: *close relationship to humans*]

Factor: knowledge

**Theoretical basis**
Deductive categories according to Chang Rundgren and Rundgren [1] and Christenson et al. [2], inductive formation of sub-subcategories:<br>*“As science educators, we always maintain the hope that students and the public could make decisions based upon scientific knowledge and evidence. The knowledge aspect could be linked to those six subject areas through the concepts, theories, laws, or evidence developed and discovered from sociology/culture, science and technology, economy, environment/ecology, policy, [...]”* [1, p. 14]<br>Statements are coded...<br>- that refer to empirical knowledge and are used to justify one's attitude.<br><br>ATTENTION: Statements that…<br>- contain word forms of "should" or words such as “if”, “as long as”, “unless”, “depends on...” are not coded under empirical knowledge, but under “conditions” (pp. VII-VIII).<br>- indicate insufficient information or underlying misinformation and are not formulated as a condition are coded under “alternative knowledge conceptions” (p. XI).

Category: sociology/culture knowledge

**Definition**	**Anchor example**
“...the socio-cultural background can be referred to not only on the larger scale of the individual's society (i.e. different religions) and culture (i.e. eastern or western), but also on the smaller scale of science community, family education, school life, and so on.”) [1, p. 12]<br>*“Students provide [knowledge about] concepts or theory from sociology/culture to support their arguments.”* [2, p. 345]<br>Statements are coded...<br>- that refer to social/cultural knowledge or social/cultural progress as a whole and are used to justify one's attitude.	*“[Animal experimentation provides] scientific and, therefore, societal progress.”* (ID 104)

Category: economy knowledge

Definition	Anchor example
"...the economic situation could be one of the concerns ... [or] the ... economic status of a country influences people ..." [1, p. 14]<br>*"Students provide [knowledge about] concepts or theory from economy subject area to support their arguments."* [2, p. 345]<br><u>Statements are coded...</u><br>- that refer to knowledge from the fields of economics and are used to justify one's attitude.	*"Furthermore, I am of the opinion that in these projects, the economic factor should not be a reason for deciding on such tests ... ."* (ID 481)

Category: environment/ecology knowledge

Definition	Anchor example
"More and more SSIs nowadays are related to environmental and ecological subject areas, such as the use of cars, nuclear power, global warming and also GMOs. According to the many SSIs that are connected to environment, we separate the environment subject area from the science subject area, although environmental science (including ecology) is one of the domains in biology." [1, p. 13]<br>*"Students provide concepts or theory from ecology/environmental science to support their arguments"* [2, p. 345]<br><u>Statements are coded...</u><br>- that refer to knowledge in the fields of ecology/environmental science and are used to justify one's attitude.	*"Behavioural studies ... can provide [information about] behaviour in nature and provide information for species conservation measures."* (ID 304)

Category: science knowledge

Definition		
"... scientific knowledge from different subjects (i.e. biology, chemistry, technology, medicine, and so on) ought to be embedded into individuals' thinking processes." [1, p. 14]<br>*"Students provide [knowledge about] concepts or theory from science (i.e. biology, chemistry, technology, medicine and so on) to support their arguments."* [2, p. 345]<br><u>Statements are coded...</u><br>- that refer to knowledge or evidence from the fields of STEM or medicine and are used to justify one's attitude.		
**Subcategory**	**Coding rule** (Statements are coded...)	**Anchor example**
Housing of test animals	- that relate to the type of housing of test animals, e.g. captivity, cages.<br>- that address the (inappropriate) husbandry of test animals.	*"I do not support animals being kept in captivity ... ."* (ID 458)

Characteristics of NHPs or other test animals	- that describe the characteristics of NHPs or other test animals.<br>ATTENTION:<br>- if two different characteristics of NHPs or other test animals are addressed within a statement, then these are coded individually.<br>- if the same characteristic is described twice within a statement, it is only coded the first time it is mentioned.		
	**Sub-subcategory**	**Coding rule** (Statements are coded...)	**Anchor example**
	Biological characteristics of NHPs	- that contain biological characteristics of NHPs, e.g. highly developed brain and thus high cognitive abilities, complex social behaviour, longer lifespan than other test animals.	*"Primates already have a well-developed brain and thus a strong capacity for sensation and memory ... ."* (ID 10)
	Biological characteristics of test animals other than NHPs	- that contain biological characteristics of test animals other than NHPs, e.g. in terms of lifespan or sentience.	*"Most other animals used for experiments have a much shorter lifespan, so conducting experiments on them leaves a smaller lasting impact on the animal itself."* (ID 6)
	Similarity humans & NHPs	- that mention similarities between humans and NHPs (in general or more specifically, such as a close genetic relationship or similar sensory perception).	*"Those animals [NHPs] are so similar to humans and it is not justified (neither ethically nor otherwise) to use these animals for our scientific advances."* (ID 19)
**Subcategory**	**Coding rule** (Statements are coded...)		**Anchor example**
Transferability of AE	that address the transferability of results from AE to humans.		
	**Sub-subcategory**	**Coding rule** (Statements are coded...)	**Anchor example**
	Problems with AE transferability	- that address problems with transferring the findings from AE to humans.	*"...results cannot always be transferred to humans without problems and therefore the animals suffer and the justification that it [animal testing] protects humans is then no longer given."* (ID 52)
	Higher transferability for AE with NHPs	- that address better transferability of findings from AE to humans when NHPs are used as test animals.	*"However, since they [NHPs] are so closely related to humans, research on them is often times directly transferable to humans."* (ID 6)
**Subcategory**	**Coding rule** (Statements are coded...)		**Anchor example**

Alternative methods to AE	- that generally refer to the existence of alternative methods to AE or refer to specific alternative methods to AE (e.g. tissue cultures, ‘in silicio’ methods or ‘body on a chip’).<br>ATTENTION: If it is stated that all AE can already be replaced by alternative methods à CODE: alternative knowledge conceptions		*“Because in many cases, such as in the early stages of drug research, tissue cultures can replace animal testing.”* (ID 11)
Lack of alternative methods to AE	- that refer to the (current) lack of alternative methods to AE.<br>- that refer to the need for increasingly looking for alternative methods to AE.<br>ATTENTION: If it is stated that there are no alternative methods to AE à CODE: alternative knowledge conceptions		*“I think that animal testing gives us many opportunities in our lives and [there] are currently no good alternatives.”* (ID 22)
Scientific findings	- that refer to the acquisition of knowledge or scientific progress achieved through AE or behavioural research.<br>ATTENTION: If the purpose of gaining scientific knowledge is explained/contextualised in more detail à classification in the following sub-subcategories:		
	**Sub-subcategory**	**Coding rule** (Statements are coded...)	**Anchor example**
	Findings in basic research<br>Definition:<br>*“Basic research projects that are initially only aimed at elucidating life processes ... can result in medical benefits at a later date.”* (DFG, 2016, 18)	- that state as their purpose the study of basic life processes in humans and/or animals.<br>e.g. research into the functioning of organs, immune and nervous systems, or the functioning of biological processes in general.	*“Animal experimentation is nec[es]sary in order to do research on organ systems and physical reactions to possible medication/vaccines.”* (ID 57)
	Findings in applied/biomedical research<br>Definition:<br>*“Research directly related to human or animal diseases”* [3, p. 10]	- that state as their purpose the study of diseases, the development of diagnostic or therapeutic methods, the development of vaccines, antibiotics or other medicines.<br>- that relate scientific findings to the specific research field of (bio)medicine or name words such as ‘medicine/medical’ in connection with scientific findings.	*“They [animal experiments] [are] helpful [in] curing diseases, developing drugs and testing vaccines.”* (ID 237)

Findings in regulatory research<br>Definition:<br>*"... test animals are used for such safety tests, quality controls or toxicological tests in accordance with chemical, pharmaceutical or food hygiene legislation."* (DFG, 2016, 11)	- which state the purpose of testing or checking the effect of medicines, drugs, chemicals, etc.	*"Without animal [experimentation], it would be difficult to determine the safety and efficacy of new drugs and procedures before they are tested on humans."* (ID 516)

Category: policy knowledge

**Definition**		
"... SSI decisions according to the policy or law made by the government." [1, p. 14]<br>*"Students provide [knowledge about] concepts or theory of policy to support their arguments"* [2, p. 346]<br>Statements are coded...<br>- that refer to political or legal guidelines, regulations and laws to justify their own attitude.		
**Subcategory**	**Coding rule** (Statements are coded...)	**Anchor example**
Laws & regulations on AE/behavioural research	- that concern generally applicable laws, regulations, provisions for the implementation of AE/behavioural research<br>- that refer to laws or regulations conceptually, e.g. Animal Welfare Act.	*"The experiment[s] with animals have strong [a]ssumptions and are strictly controlled, which is why I think it is responsible."* (ID 34)
3R principle<br>Definition:<br>*"3R stands for [R]efinement (refinement), [R]eduction (reduction) and [R]eplacement (avoidance). The aim of the principle is to avoid animal experimentation wherever possible, to reduce their number and to limit the suffering of animals in experiments to the absolute minimum."* (DFG, 2016, 48)	- that relate to the 3R principle as a rule for the implementation of AE.	*"Animal experimentation should always follow the 3R rule (Reduce, Refine, Replace)."* (ID 28)

Factor: conditions

**Theoretical basis**		
Inductive coding:<br>"*A conditional sentence [conditional clause] is used to express that a specific fact occurs under the condition that a specific other fact exists or occurs. [...] In terms of tense and mode, conditional clauses are not fixed.*" [4, pp. 335–336]<br><u>Statements are coded...</u><br>- that connect conditions to their attitudes. <u>Example:</u> *"And it does play a role what kind of product is being tested in those animal experimentation. For example, I think [it is] right to test drugs against a disease on animals, while it is wrong to test animals for a beauty product."* (ID 89)<br>- that contain the word forms of "shall/should" or words such as "if", "as long as", "unless", "depends on" ...		
**Category**	**Coding rule** (Statements are coded...)	**Anchor example**
Necessity of research	- that address attitudes depending on the perceived necessity of the research.<br>- that put words such as "necessary" or "relevant" in a conditional context with the implementation of AE/behavioural research.	*"In my opinion, the experiments conducted should be really necessary and the research question should be important/significant."* (ID 113)
Benefits of research	- that generally relate to the gain of scientific knowledge in a conditional context with the implementation of AE/behavioural research.<br>- that include that the research "should be meaningful/important" or "there should be a reason" for AE/behavioural research.	*"I am in favour of animal experimentation as long as it benefits humanity in a meaningful sense, ..."* (ID 134)
"if no other option"	- that state that AE/behavioural research are only acceptable if no other way of gaining knowledge is possible.	*"If it [AE] can help people and other living beings or provide information on issues in this direction, this is fine as long as there is no other way [of] obtaining this knowledge ... ."* (ID 77)
Rules & regulations for research	- that state that AE/behavioural research are only acceptable as long as rules or regulations for the implementation of AE/behavioural research are followed.	*"I think animal experimentation, as long as they take place under certain conditions and under certain rules with consideration of the ethical aspects, are important[,] in order to gain new knowledge in biology."* (ID 137)
Type of research	- that specifically address the types of experiments that are supported or rejected.<br>- that describe that the attitude depends on the type of experiment	*"If the [nonhuman] primates are not examined with computers which are connected to the[i]r brain by an operation I do not have a problem with behavioural biology."* (ID 30)

Effects of research on animals	- where the attitude depends on the consequences for test animals (pain, suffering, harm) because of their use in research.	*"If only the behaviour and reactions of the animals are observed and examined, I think that's fine. As soon as the animals are injured, it's no longer okay for me."* (ID 38)
Conditions under which the animals are kept	- where the attitude depends on the conditions under which the test animals are kept.<br>- that describe the species-appropriate husbandry of animals as a necessity.<br><br>ATTENTION: if named in connection with the place of the research (e.g. wilderness or laboratory) à CODE: Place of research	*"Furthermore if the [nonhuman] primates are kept approp[ri]ate to the species I think it is a good thing to analy[z]e their behaviour."* (ID 30)
Place of research	- in which the location of the experiment (e.g. wilderness or laboratory) is explicitly addressed as a condition for the implementation of AE/behavioural research.	*"It depends on the method, if they do research on nonhuman primates in the wild e.g. watch them from a safe distance and observe how they react on specific items, then I am in favor of behavioural biology. I am against all experimentation on animals in labs, in particular when they get harmed and put inside cages to get used for human research."* (ID 16)

Factor: ethical aspects

**Theoretical basis**		
Deductive subcategories according to Hölker et al. [5] inductive formation of sub-subcategories:<br>*"[In] animal-ethical arguments, two consecutive questions are usually pursued. First, it is necessary to clarify whether animals must be morally considered in human actions at all (moral status) and how this can be justified. ... Building on this, it is necessary to answer the question of what humans are allowed to do with animals (moral action)."* [5, p. 301]<br>Statements are coded...<br>- that relate to the moral status of animals or the moral treatment of animals		
**Category**	**Coding rule** (Statements are coded...)	**Anchor example**
Moral status of animals<br>Definition:<br>*"The concept of [moral status] aims at the axiological assumptions about which (kinds of) beings it is*	- that refer to the moral status of animals in general or in comparison to humans.<br>- that refer to the assigned value of animals in comparison to	

appropriate to ask why they are being treated morally rightly or wrongly.” [6, p. 392]	humans.	
**Subcategory**	**Coding rule** (Statements are coded...)	**Anchor example**
Equal moral status<br>of humans & animals	- that ascribe the same moral status to humans and animals.<br>- that consider humans and animals to be the same and see no significant differences between them.	*“In general, however, such experiments should be kept to a minimum, since the sentience of nonhuman organisms is also important to me; after all, we are animals too, so why apply different standards from those of our fellow species, unless absolutely necessary?”* (ID 2)
Equal moral status<br>of humans & NHPs	- that ascribe the same moral status to humans and NHPs.<br>- that consider humans and NHPs to be the same and see no significant differences between them.	*“Those animals [NHPs] are so similar to humans and it is not justified (neither ethically nor otherwise) to use these animals for our scientific advances. They feel the pain we feel, so why would we object them and not us to the experiments?” (*ID 19)
Equal moral status<br>of all animals	- that ascribe the same moral status to all animals.<br>- that consider all animals to be the same and do see no significant differences between them.	*“I think that the same arguments for and against animal experimentation on all animals other than primates should apply as apply to primates, because there is no obvious reason to ascribe a different moral status to primates and other animals.”* (ID 11)
Lower moral status<br>of other animals than of NHPs	- that assign a lower moral status to test animals other than NHPs than to NHPs.<br>- that do not equate other test animals with NHPs but attribute a higher value to NHPs than to other test animals.	*“I also have compassion for other test animals, but less than for nonhuman primates, probably because they are evolutionarily less close to me ... .”* (ID 9)
Lower moral status<br>of animals than of humans	- that generally assign animals a lower moral status than humans.<br>- that do not equate animals with humans in general but attribute a higher value to humans and/or human life.	*“In order to help people and expand human knowledge, it is justifiable in my opinion to harm other animals.”* (ID 135)

Lower moral status of NHPs than of humans	- that assign NHPs a lower moral status than humans<br>- that do not equate NHPs with humans, but attribute a higher value to humans and human life	*"I understand that monkeys are needed for some experiments, it's hard to experiment on humans [...]."* (ID 113)
**Category**	**Coding rule** (Statements are coded...)	**Anchor example**
Moral treatment of animals<br><u>Definition:</u><br>*"The answer to the fundamental question of animal ethics 'What are we allowed to do with animals?' can range from everything to nothing at all."* [5, p. 301]	- that refer to the moral treatment of animals in general or of specific animal species	
**Subcategory**	**Coding rule** (Statements are coded...)	**Anchor example**
Treating animals with dignity	- that state that animals should generally be treated with dignity/respect or have a right to be treated with dignity/respect.<br>- that state that the dignity of animals must not be violated.	*"Despite all the scientific achievements that can be obtained with it [AE], it [AE] is therefore irresponsible for me, because every living being should be treated with dignity."* (ID 10)
Not harming animals	- that generally reject or prohibit the infliction of pain, suffering, harm on animals.	*"I don't think we can hurt animals just for our knowledge."* (ID 58)
Not harming more highly developed animals	- that generally reject or prohibit the infliction of suffering, pain and/or harm on higher developed animals.	*"But I have something against invasive experiments since it is not okay to harm "higher developed" animals."* (ID 33)
Not instrumentalising animals	- that state that animals must not be instrumentalised, i.e. used for the purpose of humans.	*"[W]e should not utilize animals for human research."* (ID 16)

Factor: alternative knowledge conceptions

**Theoretical basis**		
Inductive coding<br><u>Statements are coded...</u><br>- that indicate insufficient information or underlying misinformation and that are not formulated as a condition.		
**Category**	**Coding rule** (Statements are coded...)	**Anchor example**
Use of AE in the cosmetics industry<br><u>Explanation:</u><br>*"Animal testing to test cosmetics has been banned in Germany since 1998. Since 2004, animal testing for finished cosmetic products has no longer been permitted within the EU. Since July 2013, there has also been a restriction that no animal testing may be carried out within the EU to test the ingredients of cosmetics."* [7, p. 73]	- where the respondent rejects AE solely based on its use in cosmetics industry.	*"For cosmetics, for example, I see no need to use animal testing and for the most part I also pay attention to whether the cosmetics I use/buy have been tested on animals."* (ID 41)
Misinformation	- that refer to misinformation about AE/behavioural research e.g. about the inexistence of laws, no alternative methods, lack of scientific findings from AE/behavioural research	*"... we've learnt that many experiments on animals are done without any real goal."* (ID 474)

Factor: personal experiences

**Theoretical basis**		
Inductive coding:<br>*"Experience (...) [is] the knowledge gained through (mostly repeated) [own] perception and experience (perception, observation, sensation)"* [8].<br>Statements are coded...<br>- that refer to personal experience(s) or describe points of contact with AE/behavioural research.		
**Category**	**Coding rule** (Statements are coded...)	**Anchor example**
Personal experiences	- that describe personal experience(s) in connection to AE or behavioural research in which the participant was directly involved.	*"I regret and do not regret any of the experiments I carried out, as they were all necessary[,] in order to save lives and gain new insights into medicine."* (ID 14)
Points of contact	- that refer to things or experiences that close people of the test person had.<br>- that refer to the test persons' own points of contact with AE or behavioural research, such as taking medication developed through AE.	*"For me it is difficult to draw a line here [AE], because the progress that animal experimentation can bring is enormous and I have already had contact with people who would not have survived without previous animal experimentation."* (ID 10)

References

1. Chang Rundgren SNC, Rundgren CJ. SEE-SEP: From a separate to a holistic view of socioscientific issues. Asia-Pacific Forum on Science Learning and Teaching. 2010;11(1).
2. Christenson N, Chang Rundgren SN, Höglund HO. Using the SEE-SEP model to analyze upper secondary students' use of supporting reasons in arguing socioscientific issues. Journal of Science Education and Technology. 2012;21(3):342–352. https://doi.org/10.1007/s10956-011-9328-x
3. Treue S, Stilling R, Berg L. [Compass Animal Testing 2024: Depict. Classify. Explain.] Kompass Tierversuche 2024: Abbilden. Einordnen. Erklären. Berlin: Initiative Tierversuche verstehen; 2024. https://doi.org/10.17617/1.0ZHW-XT44
4. Eisenberg P. [The sentence. An outline of German grammar.] Der Satz. Grundriss der deutschen Grammatik. 4th ed. Stuttgart: JB Metzler; 2013.

5. Hölker S, Steinfath H, von Meyer-Höfer M, Spiller A. Animal-ethical intuitions in Germany: Developing a measuring instrument to capture domain-specific values in the context of the human-animal relationship. German Journal of Agricultural Economics. 2019;68(4). https://doi.org/10.52825/gjae.v68i4.2143
6. Ach JS. [Moral Status] Moralischer Status. In: Prechtl P, Burkard FP, editors. Metzler Lexikon Philosophie. 3rd ed. Stuttgart: JB Metzler; 2008. p. 392.
7. German Research Foundation (DFG) Senate Commission on Animal Experimental Research. Animal experimentation in research. Bonn: DFG; 2016. Available from: https://www.dfg.de/download/pdf/dfg_im_profil/geschaeftsstelle/publikationen/tierversuche_forschung.pdf
8. Wirtz MA. [Experience.] Erfahrung. In: Wirtz MA, editor. Dorsch Dictionary of Psychology [Internet]. Bern: Hogrefe; 2023 Feb 10 [cited 2026 Jan 25]. Available from: https://dorsch.hogrefe.com/stichwort/erfahrung
